# PLGA Nanoparticles for Ultrasound-Mediated Gene Delivery to Solid Tumors

**DOI:** 10.1155/2012/767839

**Published:** 2012-02-28

**Authors:** Marxa Figueiredo, Rinat Esenaliev

**Affiliations:** ^1^Department of Pharmacology and Toxicology, University of Texas Medical Branch, 301 University Boulevard, Galveston, TX 77555, USA; ^2^Department of Neuroscience and Cell Biology, Department of Anesthesiology, and Center for Biomedical Engineering, University of Texas Medical Branch, 301 University Boulevard, Galveston, TX 77555, USA

## Abstract

This paper focuses on novel approaches in the field of nanotechnology-based carriers utilizing ultrasound stimuli as a means to spatially target gene delivery *in vivo*, using nanoparticles made with either poly(lactic-co-glycolic acid) (PLGA) or other polymers. We specifically discuss the potential for gene delivery by particles that are echogenic (amenable to destruction by ultrasound) composed either of polymers (PLGA, polystyrene) or other contrast agent materials (Optison, SonoVue microbubbles). The use of ultrasound is an efficient tool to further enhance gene delivery by PLGA or other echogenic particles *in vivo*. Echogenic PLGA nanoparticles are an attractive strategy for ultrasound-mediated gene delivery since this polymer is currently approved by the US Food and Drug Administration for drug delivery and diagnostics in cancer, cardiovascular disease, and also other applications such as vaccines and tissue engineering. This paper will review recent successes and the potential of applying PLGA nanoparticles for gene delivery, which include (a) echogenic PLGA used with ultrasound to enhance local gene delivery in tumors or muscle and (b) PLGA nanoparticles currently under development, which could benefit in the future from ultrasound-enhanced tumor targeted gene delivery.

## 1. Introduction

To achieve successful gene therapy in a clinical setting, it is critical that gene delivery systems be safe and easy to apply and provide therapeutic transgene expression. Over the past decades, many studies using viral vectors have established the gold standard for successful gene transfer and high-level expression in target cells. However, the upcoming trend is in the development of improved methods for nonviral gene transfer, due to the considerable immunogenicity related to the use of viruses. Nonviral vectors are particularly suitable since they allow ease of large-scale production and are relatively less immunogenic. Recently, several novel nonviral vectors have been developed that approach viruses with respect to transfection efficiency. A variety of nonviral delivery systems that can be used in different clinical settings are also available and one promising direction is the development of biodegradable, echogenic nanoparticle systems that can deliver DNA (or drugs) efficiently by the use of ultrasound-mediated delivery. We will focus our discussion on PLGA nanoparticles and their promise for nucleic acid delivery *in vivo* using ultrasound-mediated gene delivery methods.

## 2. Current Sonoporation Principles

 A relatively novel strategy for gene and drug delivery enhancement is application of echogenic nanoparticles made of poly(d,l-lactic-co-glycolide) (PLGA) or derivatives in combination with relatively low-intensity ultrasound (US). This method (referred to as “sonoporation”) can induce cavitation of or near cellular membranes to enhance delivery of drugs and nucleic acids *in vitro* and *in vivo*. In general, low-intensity US can induce beneficial and reversible cellular effects, in contrast to high US intensities, which are more likely to induce cellular death. Sonoporation is an emerging and promising physical method for drug and gene delivery enhancement *in vitro* and *in vivo *[1–4]. In fact, sonoporation has several advantages over other nonphysical techniques of nucleic acid (DNA, siRNA) delivery including the ability to also deliver viruses and small molecules (reviewed in [5]). Sonoporation, however, has some limitations including penetration depth, some deep (internal) tumors may not be easily accessible by US, and tissues such as bone might interfere with the US penetration. Also, the influence of air within the lung might also impair the ability of US waves to penetrate and deliver genes in the lung. Typically, sonoporation agents (also useful as US contrast agents) can be composed of micro- or nanoparticles filled with either air or gases, which give echogenic properties, surrounded by a shell of lipids or polymeric formulations. Gas-filled lipid particles are called microbubbles (MBs), while echogenic polymeric particles can be defined as either nanoparticles (NPs) or microparticles (MPs) depending on their size. Different types of MB have been synthesized by combining different shell compositions such as albumin, galactose, lipids, or polymers, with different gaseous cores such as air, or high-molecular-weight gases (perfluorocarbon, sulphur hexafluoride, or nitrogen) and several types are available commercially (reviewed in [5]). This paper will focus on echogenic NP use in combination with US-mediated sonoporation to induce gene delivery.

**Figure 1:**
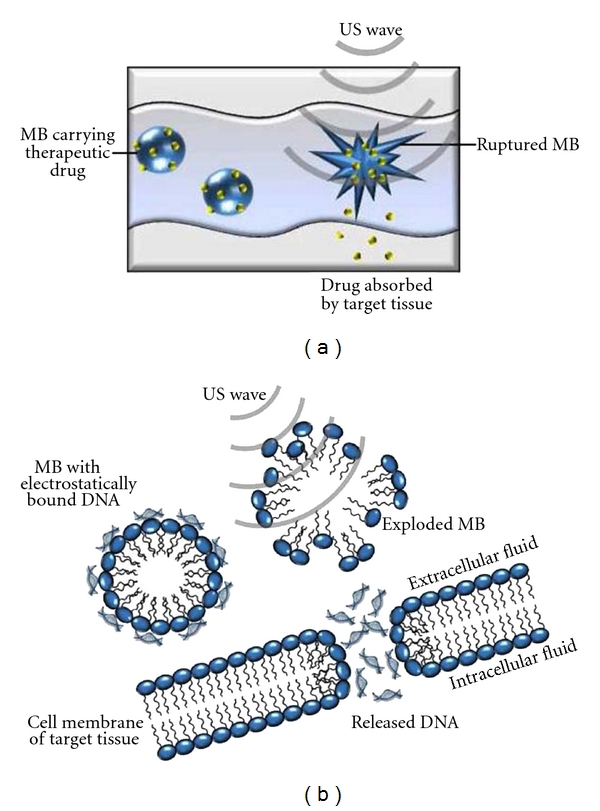
Sonoporation mechanisms for therapeutic delivery. (a) Sonoporation for drug delivery. Drugs can be delivered by sonoporation. Microbubbles with drug attached to the surface or enclosed within the particle travel in capillaries. Upon US exposure MBs rupture, releasing the drug contents. Drugs are absorbed by the target tissue. (b) Sonoporation for gene delivery. When plasmid-DNA-(pDNA-) containing MBs are passed through blood vessels adjacent to tumor cells, US waves rupture MB and release pDNA. Released pDNA penetrates into cells through their membranes by sonoporation. Reproduced with permission from [5].

The mechanism of sonoporation involves the motion of MB or NP and disruption induced by low-intensity US sonication (Figure 1). US increases the permeability of cell membranes and the endothelium, thus enhancing therapeutic uptake, and can locally increase drug/nucleic acid transport. Formation of short-lived nanopores (~100 nm) in the plasma membrane lasts a few seconds and is implicated as the dominant mechanism associated with acoustic cavitation [6]. Sonoporation mediates delivery of drugs and/or nucleic acids that have been incorporated into or on the surface of nano/microparticles via covalent or electrostatic interactions, which allow these complexes to circulate in the blood and retain their cargo until activation by US. US application results in localized and spatially controlled particle disruption that enhances gene/drug delivery. Sonoporation-mediated gene delivery has been applied to date in heart, blood vessels, lung, kidney, muscle, brain, and tumors with high efficiency [7]. However, in order to provide high transfection efficiency, ultrasonic parameters (such as acoustic pressure, pulse length, duty cycle, repetition rate, and exposure duration) and nano- or microparticle properties (such as size and echogenic characteristics of air- or gas-filled preparations) should be optimized [7]. The efficiency of drug/gene delivery typically correlates to the cell location relative to the US (transducer and its proximity to acoustically active nano- or microparticles). At ~1 MHz US, echogenic nano/microparticles or microbubbles oscillate steadily. It has been shown that lipid-shelled MB can expand from 2 *μ*m to ~20–55 *μ*m [8]. When MBs expand and collapse near a cell wall, a fluid jet/shock wave is formed followed by an increase in vascular permeability [9]. In this manner, drug or nucleic acid transport may occur by convection through a membrane pore [8], and this US-induced effect may represent the main mechanism for sonoporation-mediated gene or drug delivery. This is supported by correlation of the uptake of a dye with cellular deformation and membrane changes as assessed by scanning electron microscopy, membrane electrophysiology and atomic force microscopy [10–12]. Following pore formation, nonspecific uptake of extracellular molecules can occur, the membrane is repaired, and molecules are, therefore, retained within cells. Mammalian cells have been shown to repair pores of up to ~1000 *μ*m^2^ within a short period [13], in a manner resembling the kinetics of membrane repair after mechanical wounding, and Ca^2+^ levels are thought to promote this response [14, 15].

## 3. Echogenic Nanoparticles

**Figure 2:**
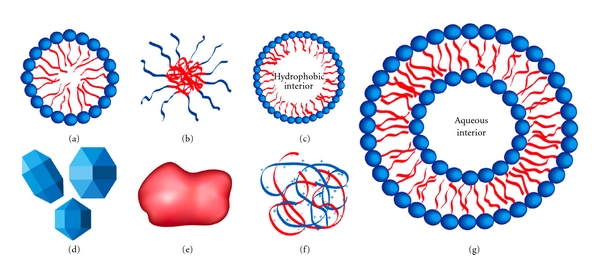
Various nanoparticles (not to scale) that may be used in ultrasound-enhanced drug and gene delivery. (a) Micelle (nonpolymeric) composed of amphiphilic surfactants. (b) Polymeric micelle composed of amphiphilic block copolymers. (c) Nanoemulsion consisting of a hydrophobic liquid core stabilized by surfactant. (d) Crystalline nanoparticles. (e) Amorphous polymeric nanoparticle. (f) Condensed ionic oligomers, such as DNA condensed with PEI or cationic lipids. (g) Single-walled liposome consisting of an amphiphilic bilayer surrounding an aqueous core. Reprinted with permission from [71].

In this paper, nanoparticles (NPs) are defined as molecules ranging in size from 1 nm to 1 *μ*m and that are able to form a separate phase in aqueous suspension. Echogenic NPs are defined as NPs containing either atmospheric air or gas to form “nanobubbles” that can be used for drug and gene delivery when US is applied. In most medical applications, NPs typically are in suspension and can be classified into micelles, nanoemulsions, and suspensions of solid nanoparticles (Figure 2). Most of them have been tested for US-mediated gene delivery.

### 3.1. Nanoparticles Used for Gene Delivery

#### 3.1.1. Lipid-Based Nanoparticles

 Complexing of cationic lipids and DNA plasmids (lipofection) is efficient at transfection of various cell lines and several lipid combinations are available commercially. However, there has been little combination of US with lipofection, possibly because early studies using ultrasound and gas bubbles showed that the addition of the contrast agents enhanced transfection of naked DNA much more than traditional transfection by lipofection, which is mediated through endocytosis and pinocytosis mechanisms [16]. The incubation time of lipofection from transfection to gene expression is also slower compared to that with naked DNA and contrast agents [17]. Of the few studies that combined US and lipofection, one example highlights the challenges of this method. For example, brain tumor cell transfection using 2 MHz pulsed US for 1 min and Lipofectamine condensed with plasmids coding for green fluorescent protein (GFP) produced no change in transfection efficiency compared to conventional lipofection alone [18]. Therefore, it appears that lipofection is not enhanced by US unless gas bubbles are introduced in the liposome or present as a separate agent. If gas bubbles are present, the transfection by naked DNA + US then appears to be effcient *in vitro*. However, there are several advantages with respect to enhanced durability when plasmids are complexed with cationic lipids. 

#### 3.1.2. Polymeric Nanoparticles

 Polymers used for drug and gene delivery typically include polystyrene (PS), poly(lactic acid) (PLA), poly(lactic-co-glycolic acid) (PLGA), and polyplexes of plasmids and cationic polymers. Application of US to solid polymeric nanoparticles appears to be effective in reducing cavitation threshold in water, even in the absence of preformed gas bubbles [19]. For example, we have shown that PS nanoparticles can reduce the threshold of US-induced cavitation activity in pure water from about 7.3 bar to <5 bar, depending upon the size and concentration used [1, 20]. We observed that the threshold decreased with increasing particle concentration and particle sizes [1, 20]. Thus, even without the use of gas bubble contrast agents, there was sufficient cavitational activity to produce significant bioeffects. Although other investigators have used other polymer and polyplex nanoparticles, they did not report whether these particles lowered thresholds or enhanced US activity. For potential translational applications, it would be very beneficial to know whether other types of solid nanoparticles can lower the cavitation threshold in blood or in intracellular liquids. 

*One important reason for selecting NP over commercially available MBs as sonoporation enhancers is the ability of NPs to extravasate in capillaries and beyond, whereas MBs cannot due to their larger dimensions.* In fact, this capability of NPs enables their efficient delivery to tumor cells, where US can then induce spatially confined cavitational activity (sonoporation) to enhance gene delivery. For example, we have shown that approach allowed for vasculature disruption only in US-irradiated tumors of nude mice, while no disruption was observed in nonirradiated controls [21]. In another study, we investigated the influence of polystyrene nanoparticles (100 and 280 nm in diameter and concentration up to 0.2% w/w) on cavitation threshold in water at the frequency of 20 kHz. Then, we studied efficacy of cancer chemotherapy with this technique *in vivo*. The experiments were performed in athymic nude mice bearing human colon KM20 tumors, which are highly resistant to chemotherapy. Ultrasound with the frequency of 20 kHz in combination with i.v. injected polystyrene nanoparticles was applied to enhance delivery of chemotherapeutic agent 5-fluorouracil [1]. Our studies demonstrated that US irradiation in combination with the NP and drug injections significantly decreased tumor volume and resulted in complete tumor regression at optimal irradiation conditions, while the volume of control (nonirradiated) tumors increased despite drug injections. These data suggest that US-induced drug delivery may improve efficacy of current cancer treatment regimens, suggesting PS + US do not cause significant tumor cell toxicity and can be used safely to deliver drugs or nucleic acids. For instance, when PS + US were used to deliver 5-FU, the antitumor effect was augmented dramatically for this drug, with a 60% growth rate reduction and enhanced necrosis throughout the tumors as observed by histology. Another *in vivo* study showed that polystyrene nanoparticles decrease cavitation threshold in water, and application of this drug delivery technique substantially improved the efficacy of cancer therapy in nude mice with colon tumors when US was used in combination with polymer NP injections [20].

**Gene Delivery by Polymeric PLGA Nanoparticles.**

Several studies have shown efficient US-enhanced gene delivery using polyplexes of DNA and cationic-derivatized natural polymers, such as cationized dextran [22] and gelatin [23]. In these experiments, 3 MHz US (2 W/cm^2^, 10% duty cycle) typically was applied for 1 to 2 minutes transdermally to various tissues *in vivo* such as tumors or muscle. Insonation always enhanced gene expression for a few days. The authors speculated that cavitation-induced cell membrane damage and permeation were responsible for the enhanced gene expression.

Arguably, superior polymeric nanoparticle formulations for gene delivery using US may be composed of PLGA, a polymer approved by the FDA for its excellent profile of biodegradability, drug biocompatibility, suitable biodegradation kinetics, mechanical properties, and ease of processing (reviewed in [24]). PLGA and its derivatives have been the center focus for developing nano/microparticles encapsulating therapeutic drugs in a biodegradable format. Many macromolecular drugs including proteins, peptides, genes, vaccines, antigens, and human growth factors can be incorporated successfully into PLGA- or PLGA/PLA-based nano/microparticles. And several microparticle formulations already are available in the market (reviewed in [25]). However, intense research is ongoing to refine and enhance PLGA-based NP over other delivery systems, including developing blends of PLGA with other polymers, for example, chitosan, pectin, poly(propylene fumarate), poloxamers and poloxamines, polypyrroles, gelatin, poly(vinyl alcohol) (PVA), PVA-chitosan-PEG, and poly(ortho-esters) (reviewed in [25]). These novel technologies can produce PLGA- and PLGA-based nano/microparticles for drug delivery and can dramatically expand the new field for efficient drug/gene delivery if the nanoparticles can be rendered echogenic or acoustically active.

Biodegradable PLGA NPs can sustain delivery of drugs, proteins, peptides, and plasmid DNA, owing to their ability to protect macromolecules from degradation in endolysosomes (reviewed in [26]). NPs have distinct advantages for drug delivery since they can penetrate deep into tissues through fine capillaries, across fenestrations present in the epithelial lining and, generally, are taken up efficiently by the cells, allowing efficient delivery of therapeutic agents. NPs also have the advantage of sustaining the release of the encapsulated therapeutic agent over a period of days to several weeks compared with natural polymers that have a relatively short duration of drug release [27]. The safety of PLGA-based NPs in the clinic has been well established [28] and polyethylene-glycol- (PEG-) conjugated PLGA NPs are currently emerging as molecules with reduced systemic clearance compared with similar NPs lacking PEG [29]. Therefore, the field of gene delivery will continue to refine and expand into PLGA NP for *in vivo* use, particularly with US-mediated enhancements in efficiency.

**Defining Sonoporation Parameters for Successful Gene Delivery Using NP.**

Efficacy and safety of cancer chemo- and biotherapy are limited by poor penetration of anticancer drugs from blood into tumor cells. Tumor blood vessel wall, slow diffusion in the interstitium, and cancer cell membrane create significant physiological barriers for macromolecular agents. We have used nano- and microparticles in tumors followed by ultrasound-induced cavitation for safe and efficient drug and gene delivery. In several studies, sonoporation has effectively enhanced anticancer drug or gene delivery in tumor cells and tissues. In our experience, sonoporation does not appear to negatively impact cellular viability of insonated tumor cells or normal surrounding tissues after treatment with either chemotherapeutic drugs [2] or plasmid DNA *in vitro* [30] or *in vivo* [4] when MBs are utilized as the gene carrier (Optison or SonoVue). SonoVue is an ultrasound contrast agent made of MB stabilized by phospholipids and containing sulphur hexafluoride (SF6), an innocuous gas [31] and manufactured by Bracco Diagnostics Inc, USA. Optison is an ultrasound contrast agent, consisting of gas-filled MBs surrounded by a solid shell of heat-denatured human albumin [32] resulting in a size range of 2.0 to 4.5 *μ*m and manufactured by GE Healthcare, USA. For example, we have shown minor damage to MCF-7 breast cancer cells following exposure to low-intensity US in the presence of either Optison MB or a chemotherapeutic drug, 5-fluorouracil (5-FU) as assessed by low lactate dehydrogenase (LDH) release (a measure of cytotoxicity) and MTT cell viability assays. However, depending on the US parameters chosen, temperature changes can be observed *in vitro*. For example, increases in US duty cycle enhanced cell death associated with either Optison or 5-FU, using 3 MHz and 2 W/cm^2^ for 1 min, while temperature changes were negligible at low US duty cycles (5%). When a duty cycle of 20% was used, heating occurred from 18°C to 36°C, while, at a duty cycle of 50%, heating rose up to 40°C. Optison at 10% appeared to protect cells from the US heating bioeffects. Cell viability was decreased by Optison dramatically when a 50% duty cycle was used and augmented by 5-FU delivery. Therefore, careful selection of US parameters is required to avoid any heating and cell toxicity. Interestingly, immediately after treatment, cell death was most dependent on Optison; however, 24 h after treatment, cell death was more dependent on 5-FU, and the best minimal effective dose for cell killing was 10 *μ*g/mL. Furthermore, treatment with 5-FU and US increased the levels of Bax and p27^kip1^ proapoptotic proteins, but the addition of Optison appeared to suppress apoptotic protein expression. This study clearly illustrates the need for experimental design aimed at dissociating specific from nonspecific toxicity effects of a gene or drug delivered by sonoporation in order to better refine the conditions for delivery *in vivo*. Another detailed study that illustrated the importance of examining the best parameters for delivering macromolecules used a macromolecule that modeled the M_w_ of drugs or plasmid DNA and delivery with Optison [1, 30], whereby transfection was obtained up to ~37% with minimal cell death, identifying optimal parameters of US exposure able to produce efficient delivery of macromolecules.

Like MBs, in our experience, echogenic nanoparticles made from polystyrene (PS) or PLGA also do not appear to produce any toxic effects in the presence of US. For example, in an *in vivo* DU145 prostate cancer model, no alterations are seen histologically to indicate cell death in tissues for PLGA NP plus US, even in the presence of pDNA:PEI complexes [3]. The next section will cover in detail strategies for US-mediated DNA delivery with PLGA and PEI:pDNA NP *in vivo*.

#### 3.1.3. Ultrasound Enhances Gene Delivery by PLGA When pDNA Is Complexed with Polycationic Polymers

Over the years, a significant number of cationic polymers have been explored as carriers for gene delivery (reviewed in [33]) since they condense DNA into small particles and facilitate uptake by endocytosis. One of these cationic polymers is poly(ethylene imine) or PEI (reviewed in [34]). The potential of PEI was first described for gene delivery applications in 1995 [35]. Several molecular weights of PEI have been investigated with the most suitable forms ranging in 5–25 kDa [36, 37]. Higher-molecular-weight PEI increases cytotoxicity due to polymer aggregation at the cell surface [38]. Low-molecular-weight PEI is less toxic yet is usually less effective for gene delivery, since the lower amount of positive charges per molecule makes it difficult for small PEIs to appropriately condense negatively charged DNA molecules. Gene delivery research has used either hyperbranched or linear PEI, and branched PEI has shown stronger complexation with DNA since it typically forms smaller complexes DNA:linear PEI [39]. The condensation behavior of branched PEI:DNA is less dependent on buffering than high-molecular-weight PEI, yet the transfection efficiency of linear PEI (22 kD):DNA complexes is typically higher than that of branched PEI (25 kD) when prepared in a salt-containing buffer [39]. * in vivo*, linear PEI:DNA complexes prepared in high salt conditions are 100-fold less active than complexes prepared in low salt conditions, suggesting efficient transgene expression depends greatly on the size of DNA complexes.

**Figure 3:**
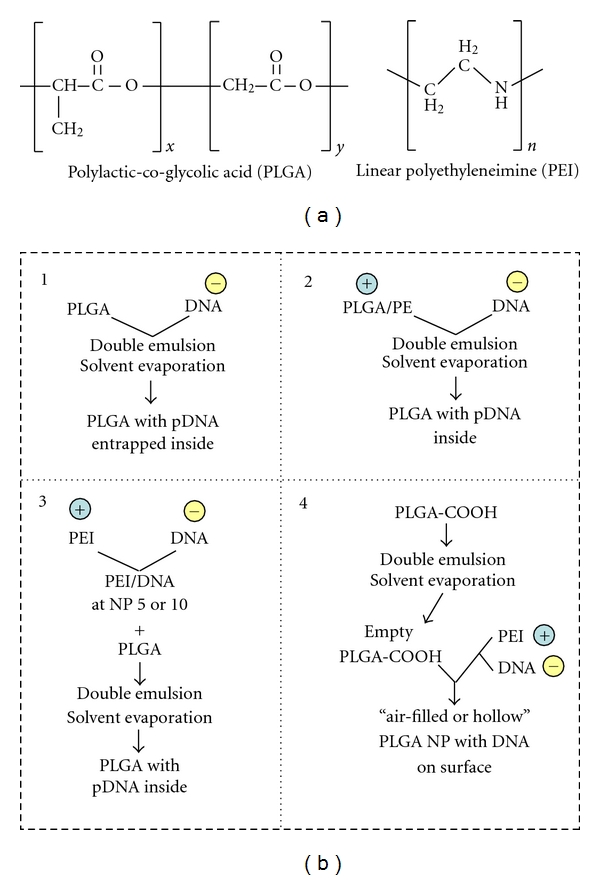
Different strategies to complex DNA with PLGA-based nanoparticles. (a) Structure of polylactic-co-glycolic acid (PLGA) and linear polyethylenimine (PEI). A branched PEI can also be utilized to form complexes. (b) Schematic of the preparation methods of PLGA formulations using methods that result in plasmid DNA incorporation on the inside or the surface of PLGA particles as reported in [3, 40].

Recently, we have shown that linear PEI (*in vivo* JetPEI) can enhance echogenic PLGA NP plasmid DNA (pDNA) delivery *in vivo* with US. Several ways exist to produce PLGA:PEI:pDNA particles from the original PLGA structure and branched or linear PEI molecules and these are depicted. The order in which PLGA particles are formulated with polycation PEI appears to affect gene expression magnitude. For example, Zhang et al. (Figure 3(a)) have compared three formulation methods for preparing microparticles containing PLGA PEI and pDNA and evaluated the methods for buffering capacity, cellular uptake, transfection efficiency, and toxicity. In the first method, PLGA PEI pDNA microparticles are prepared by entrapping pDNA in blended PLGA/PEI using the double emulsion water-in-oil-in-water solvent evaporation technique (PA) [40]. In a second approach, PEI-pDNA polyplexes are prepared and then entrapped in PLGA microparticles using a double emulsion solvent evaporation method (PB). Microparticles prepared using formulation methods PA and PB are then compared against PLGA microparticles with PEI conjugated to the surface using carbodiimide chemistry (PC); 0.5% PVA is identified as the optimum concentration of surfactant for generating the strongest transfection efficiencies. N : P ratios of 5 and 10 are selected for preparation of each group. Gel electrophoresis demonstrated that all PLGA formulations had strong pDNA binding capacity with significantly lower *in vitro* cytotoxicity for PLGA PEI microparticles than for PEI alone. PLGA PEI pDNA microparticles mediate higher cellular uptake efficiency and consequently higher transgene expression than unmodified PLGA microparticles in COS7 and HEK293 cells.

 Preparing PEI-pDNA polyplexes prior to entrapment in PLGA microparticles (PB) results in a higher pDNA loading capacity than pDNA loaded onto unmodified PLGA microparticles. PLGA PEI pDNA microparticles prepared in this manner and with a N:P ratio of 5 provide the strongest transfection efficiency, which is ~500-fold and ~1800-fold higher than that obtained with unmodified PLGA pDNA microparticles in HEK293 cells and in COS-7 cells, respectively (Figure 3(a)) [40]. One downside of this formulation strategy is that the particles generated are in the micron range, limiting systemic *in vivo* use. This study, however, guided our rationale for developing improved PLGA:PEI:pDNA particles, whereby strategy refinement was achieved by producing instead echogenic nanoparticles of PLGA. For our studies, we selected linear PEI (LPEI) (Figure 3(b)) [3] since it is reportedly less toxic to cells than its branched counterparts, perhaps due to mediating a lower condensation of LPEI:DNA complexes and a more efficient intracellular dissociation following uptake. LPEI:DNA complexes have been shown to enter the nucleus more readily than branched PEI:DNA [39].

**Figure 4:**
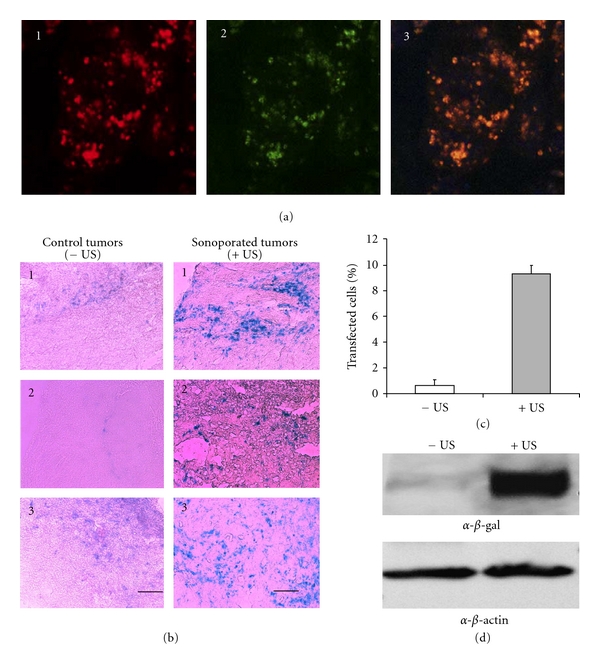
PLGA nanoparticles deliver plasmid DNA efficiently *in vitro* and *in vivo*. (a) *In vitro delivery*: cellular internalization in calu-3 cells 6 h after application of PLGA-PEI nanoparticles loaded with rhodamine-labeled GFP encoding plasmid DNA. (1) Immunofluorescence of anti-lysosomal-associated-membrane-protein-1 (LAMP-1) (red), (2) intracellular distribution of rhodamine-labeled DNA (green), and (3) superimposition of the confocal micrographs indicating colocalization of the DNA in the lysosomal compartments (orange-yellow). Reproduced with permission from [41]. (b) *In vivo delivery*: a special formulation, PLGA:PEI:DNA is excellent for I.V. gene delivery *in vivo.* (a) Bgal expression in control (left) and ultrasonicated (right) tumors with PLGA/PEI/DNA complex nanoparticles injected intravenously. Light scattering microscopy images taken at 20x; bar represents 100*μ*m. ~10% of tumor cells are transfected in ultrasonicated tumors compared to controls (*P < 0.01). Reproduced from [3] with permission from Elsevier. (c) Percentage of B-galactosidase-positive (Bgal+) cells in DU145 tumors in the absence of ultrasound (−US) and presence of US (+US). *P < 0.05. (d) Western blot showing the levels of B-gal protein are higher in tumors that received US (+US) compared to those without US treatment (−US). Levels of B-gal are shown relative to those of a control housekeeping protein, beta-actin.

The PLGA:PEI:pDNA complexes shown in Figure 3(b) -(4) are effective in delivering genes to the lung (Figure  4(a)) and prostate tumors when ultrasound is applied (Figure  4(b)). Pulmonary gene delivery can be an excellent route for gene therapy of lung-related genetic diseases and may induce immunity towards pathogens entering the body via the airways. For example, PLGA NPs prepared bearing polyethyleneimine (PEI) on their surface were characterized for their potential to transfect the pulmonary epithelium [41]. These particles were synthesized at different PLGA-PEI ratios and loaded with DNA in several PEI-DNA ratios, exhibiting narrow size distributions, with mean particle sizes ranging from 207 to 231 nm. Zeta potential was strongly positive (>30 mV) and loading efficiency high (>99%). Internalization of the pDNA-loaded PLGA-PEI NP was examined in the human airway submucosal epithelial cell line, Calu-3, and gene expression was detected in the endo-lysosomal compartment as soon as 6 h following application of particles (Figure  4(a)). NP cytotoxicity was dependent on the PEI-DNA ratio and the best cell viability was achieved by PEI-DNA ratios of 1 : 1 and 0.5 : 1. Although this example did not use US to mediate gene delivery, it illustrated the potential of PLGA-PEI NP for achieving lung epithelium transfection as well as the importance of carefully titrating the ratio of PEI to pDNA in order to not exacerbate this cationic polymer toxicity effects.

In our *in vivo* studies with similar PLGA:PEI:pDNA NP, we have shown that polyplexes of *β*-gal reporter gene plasmid DNA and linear polyethyleneimine derivative (*in vivo* JetPEI) can be formed and complexed with ~200 nm echogenic PLGA NP [3]. PLGA:PEI:pDNA complexes were administered into DU145 prostate tumor-bearing nude mice and, immediately after, a low-intensity US was applied to the tumor site. Pulsed insonation for 5 minutes at 1 MHz and −7 bars produced a significantly greater expression of the reporter gene in the tumor (~10% cells are positive for the reporter gene LacZ) compared to the noninsonated bilateral control tumor (~1% cells positive for LacZ gene) (Figure 4). Therefore, US augmented gene delivery *in vivo*. One important component of these studies was the echogenic property of the PLGA nanoparticles. These particles were prepared in a manner that resulted in “air-filled” particles that were able to oscillate in the acoustic field, which likely stimulated their or DNA uptake by endocytosis. The particles zeta potential was 13.4 ± 2.6 mV, and echogenicity properties were tested using ultrasound imaging, which revealed a similar acoustic activity as standard Definity microbubble particles. Definity particles are lipid-encapsulated microbubbles containing perfluoropropane gas ranging in size from 1.1 to 3.3 *μ*m [42] and manufactured by Bristol-Myers Squibb Medical Imaging, US. The overexpression of the *β*-gal reporter gene delivered was examined by X-gal staining and Western blot, and at least an 8-fold increase was observed in cell transfection efficiency in irradiated tumors compared to nonirradiated control. Negligible cell death was produced by ultrasonication and we determined the pDNA condensed by PEI was protected from degradation even under US conditions. These results indicated that this formulation is promising for *in vivo* gene delivery of plasmid DNA using sonoporation. PLGA and PEI each are formulation choices that have certain advantageous chemical and structural characteristics that can enhance pDNA delivery in tumor cells. The advantage of PLGA, as discussed earlier, is the biodegradability profile and echogenicity of the prepared NP. The advantage of the *in vivo* jetPEI, as shown by our data, was its ability to protect pDNA from any potential US-induced damage. Also, PEI could further enhance NP translation potential as this polymer already has been utilized in clinical trials for bladder cancer [43]. Moreover, an important rationale for using PEI to condense pDNA and complex it to the surface of echogenic PLGA NP is to enable delivery of a large amount of pDNA (≥50 *μ*g) [3], which is usually necessary to achieve efficacy in *in vivo* gene therapy settings [4], while still preserving the nanoscale dimensions of the chimeric NP (~200 nm). In some cases, pDNA can be loaded inside the PLGA NP, but usually this results in minimal encapsulation (5%) for this NP type, requiring a microparticle production. For example, IL-10 is an anti-inflammatory molecule that has achieved interest as a therapeutic for neuropathic pain. In one recent study, encapsulation of plasmid was low (only ~8 *μ*g pIL-10) when PLGA microparticles of ~4.6 *μ*m were utilized to deliver IL-10 [44]. And although this PLGA:pIL-10 therapy was able to relieve neuropathic pain for greater than 74 days in an animal model following direct intrathecal administration, a micron-sized particle such as this may be less desirable for tumor therapy and targeting, for example, as penetration and retention into tumor vasculature is desired with or without using sonoporation for gene delivery. However, refinements are possible that will allow incorporation of other choices of cationic polymers for DNA condensation and loading onto echogenic PLGA NP for further reductions in any potential PEI *in vivo* toxicity [38, 45], and potential approaches will be discussed as follows.

 Another polycation that would potentially be useful for condensing pDNA while enhancing US-mediated gene delivery is poly(L-lysine) or PLL, which has been used widely in gene therapy studies. One interesting recent study has shown that improvements can be made to PLL to reduce cytotoxicity and enhance transfection efficiency. This more efficient polymer is composed of short oligolysine grafts strung from a hydrophobic polymer backbone [46] and gives transfection efficiency greatly superior to PLL. The oligolysine graft length was altered to improve DNA-polymer interactions and overall transfection efficiency. Additionally, when PKKKRKV heptapeptides (the Simian virus SV40 large T-antigen nuclear localization sequence) were added onto the oligolysine polymer backbone, transfection efficiency was further enhanced and reporter gene expression levels reached levels higher than, or comparable to, JetPEI, FuGENE 6, and Lipofectamine 2000, the latter being notorious for cytotoxicity accompanying high transfection efficiency. Using heparin decomplexation assays, the mechanism for the enhanced gene delivery was determined to involve the relative strength of the polymer-DNA complex, contributing to the therapeutic promise of these novel oligolysine reagents since they are able to better release DNA during the transfection process following nuclear uptake.

**Figure 5:**
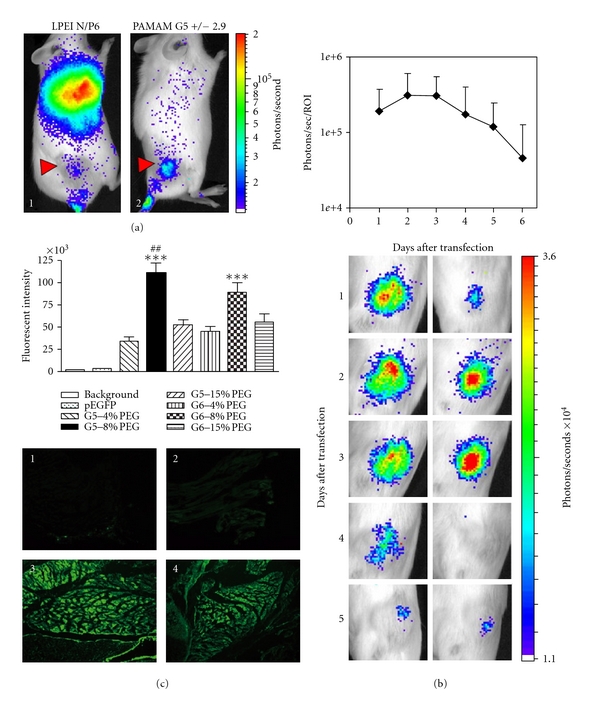
PAMAM-dendrimer-based complexes may be an alternative to PEI for pDNA delivery *in vivo* using NP. (a) PLGA:PAMAM-G5 gives higher tumor expression of reporter pDNA and lower nonspecific lung transfection for a more favorable biocompatible profile *in vivo*. In this example, A/J mice with subcutaneous Neuro2A tumors received a single intravenous injection of LPEI polyplexes N/P 6 (1) or PAMAM-EDA G5 polyplexes charge ratio 2.9/1 (2) containing plasmid pCpG-hCMV-Luc (2.5 mg/kg based on pDNA) and BLI was carried out 24 h later. Reprinted with permission from [47]. (b) Prolonged reporter gene expression in Neuro2a following intravenous administration of pCpG-hCMV-Luc/PAMAM-EDA G5 polyplexes (± 2.9). Reprinted with permission from [47]. (c) PLGA:PAMAM G5-PEG nanoparticles deliver plasmid DNA more effectively (muscle) than G5. Reprinted with permission from [54].

 Another potential DNA condensation agent for high-level gene delivery would involve the use of dendrimers of poly(amidoamine) or PAMAM. These have several advantages over PEI *in vitro* and *in vivo*, including a lower toxicity profile and reduced nonspecific lung transfection. An interesting recent study has shown that pDNA condensed with PAMAM starburst dendrimers (generation 4 and 5) can efficiently transfect tumor cells *in vitro *and *in vivo* [47]. Following intravenous injection of polyplexes into immunecompetent mice bearing subcutaneous, well-vascularized murine neuroblastoma (Neuro2A), luciferase reporter gene expression was detected predominantly in the tumor, while negligible transgene expression levels were detected in other organs as determined by bioluminescent *in vivo* imaging (BLI) (Figure  5(a)). Compared to linear PEI (LPEI), Luc expression was relatively higher and lung signals were greatly reduced for PAMAM-G5:pLuc, indicating this is a promising polyplex for *in vivo* gene delivery to tumors. Additionally, repeated applications of this polyplex type were well tolerated and resulted in prolonged average transgene expression in tumors as determined by BLI (Figure  5(b)). Fluorescence *in vivo* imaging using these polyplexes labeled with near-infrared emitting semiconductor quantum dots revealed that, although lung accumulation was similar for both PAMAM and LPEI polyplexes, only LPEI polyplexes induced high luciferase expression in lung. The mechanism proposed may involve aggregation of LPEI:pDNA with blood components that can induce backpressure in the blood flow, pushing plasmid through the lung endothelium into the vicinity of alveolar cells. Alveolar type II pneumocytes, beside endothelial cells, comprise the major fraction of transfected cells following of LPEI:pDNA i.v. injection. Therefore the authors concluded that although PAMAM polyplexes were trapped within the lung due to charge interactions, the occlusion of capillaries might not be effective enough to induce effects similar to LPEI in lung, and transfection signals are not detectable. At any rate, the PAMAM-G5 dendrimer could be a potential candidate for loading pDNA onto echogenic PLGA NP since, as PEI, it promises to have highly desirable characteristics of enhanced gene delivery that is restricted to tumors and a reduced off-target (lung) reporter gene expression *in vivo*. Finally, another promising new cationic polymer that could be a great candidate for complexing with PLGA is one containing a branched oligoethyleneimine (OEI, 800 Da) core, diacrylate esters as linkers, and oligoamines as surface modifications [48]. Although complex in structure, these are also promising since they exhibit low cytotoxicity *in vivo* and were shown to transfect tumor tissue at levels comparable to those with PEI but were better tolerated with no change in liver histology or liver enzymes, while LPEI and BPEI resulted in an increase in liver enzyme levels, suggesting early necrotic stages in liver 24 h after treatment. OEI also exhibited a more tumor-specific gene expression profile than when PEI was used, with lower lung transgene expression. Finally, dendrimers also can be used to target nucleic acid delivery to particular cells or tissues using cell-penetrating peptides. For example, PAMAM-G5 dendrimers displaying cyclic RGD targeting peptides (PAMAM-RGD) improved transport [49] and also could deliver siRNA in polyplex complexes of ~200 nm, mediating more efficient nucleic acid delivery through multicellular 3D U87 glioma spheroids than that of native PAMAM dendrimers, presumably by interfering with integrin-ECM contacts present in a three-dimensional tumor model [50]. 

 Although highly efficient nonviral gene carriers, one common drawback of LPEI, PLL, and PAMAM dendrimer cationic polymers is that these may present a high toxicity *in vivo*, even if a relatively low cytotoxicity is initially observed *in vitro*. Therefore, some solutions have included surface modification to significantly help reduce their toxicity [51–53]. For example, to help expand the *in vivo* applications of PAMAM, one study attempted to improve characteristics of this polymer as a gene delivery carrier by incorporation of polyethylene glycol (PEG, molecular weight 5,000). PEG is known to convey water solubility and biocompatibility to conjugated copolymers and usually does not adversely affect self-assembly of copolymer with pDNA, still allowing nanosized complex formation with a narrow particle size distribution. When PEG was conjugated to G5 and G6 PAMAM dendrimers (PEG-PAMAM) at three different molar ratios of 4%, 8%, and 15% (PEG to surface amine per PAMAM dendrimer molecule) [54], *in vitro* and *in vivo* cytotoxicities were reduced significantly. Also, hemolysis was reduced, especially at higher PEG molar ratios. Among all of the PEG-PAMAM dendrimers, 8% PEG-conjugated G5 and G6 dendrimers (G5-8% PEG, G6-8% PEG) were the most efficient in delivering genes to muscle following direct administration to neonatal mouse quadriceps (Figure  5(c)). Consistent with the *in vivo* results, these two 8% PEG-conjugated PAMAM dendrimers could also mediate the highest *in vitro* transfection in 293A cells. Therefore, G5-8% PEG and G6-8% PEG possess a great potential for gene delivery and could conceivably be adapted to condense nucleic acids and be loaded atop echogenic PLGA NP for US-mediated enhancements in intramuscular gene delivery.

 Other preparations successful in intramuscular gene delivery have been described, of interest since they enhance US-mediated gene delivery. These include efficient gene transfer in muscle to deliver basic fibroblast growth factor (bFGF) angiogenic gene therapy in limb ischemia. Bubble liposomes (DSPE-PEG_2000_-OMe with perfluoropropane) were used to transfect muscle in the presence of US [55]. In this example, bFGF was delivered and capillary vessels were enhanced and blood flow improved in the bFGF + MB + US-treated groups compared to other treatment groups (non-treated, bFGF alone, or bFGF + US). Skeletal muscle is a target of interest for gene delivery since it can mediate gene therapy of both muscle (e.g., Duchenne Muscular dystrophy) and nonmuscle disorders (e.g., cancer, ischemia, or arthritis). Its usefulness is due mainly to the long-term gene expression profile following gene transfer, which makes it an excellent target tissue for the high-level production of therapeutic proteins such as cytoskeletal proteins, trophic factors, hormones, or antitumor cytokines. Refining the conditions for sonoporation as well as the optimal formulation for achieving high-level transgene expression in skeletal muscle will continue to be an important focus of gene therapy delivery efforts for treating tumors, and in particular the delivery of antitumor cytokines.

#### 3.1.4. MB Can Enhance NP Gene Delivery by Sonoporation in Muscle Tissue

An interesting concept to aid NP gene delivery by sonoporation has employed combination with microbubbles *in vivo*. In one example, the hypothesis was tested that combination of a low concentration of MB could help reduce any US bioeffects and allow similar levels of transfection to occur when using PLGA NP at a lower US intensity and with a shorter duration in time. One interesting study examined the potential of improving siRNA delivery of retinal cells (RPE-J) in the presence of PLGA NP and a small amount of SonoVue MB [56]. Low-intensity US or 15–20% SonoVue MB also increased the siRNA delivery efficiency when a lower concentration of PEG and Poly-lysine-conjugated PLGA particles were used. The combination of US with MB was used to select the optimal enhancement of NP delivery but did not furhter increase the cellular uptake of NP, but it achieved significantly higher PDGF-BB gene silencing compared to NP alone.

**Figure 6:**
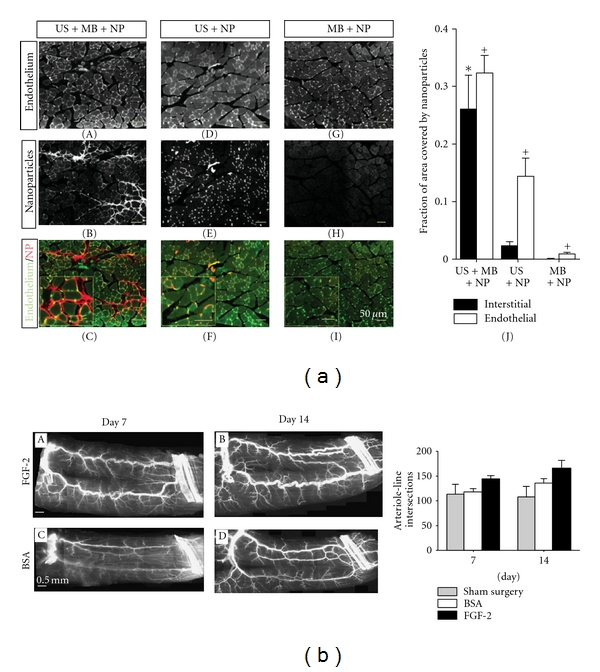
Nanoparticle uptake can be enhanced by ultrasonication in the presence of microbubbles in skeletal muscle *in vivo*. (a) Gracilis skeletal muscle cross-sections illustrating fluorescent polystyrene nanoparticle (NP) delivery for each treatment. (A)–(I) Muscle treated with ultrasound (US) + microbubbles (MB) + nanoparticles (NP) combinations. For the conditions of US + MB + NP, NPs (red) accumulate in vessel walls and muscle interstitium (BS-1 lectin staining, green). For muscle treated with US + NP, NPs colocalized with endothelium but minimal interstitial deposition was observed. Muscle treated with MB + NP was almost void of NP. (J) Bar graph representing the fraction of interstitial area (regions outside of muscle fibers and vascular structures) or endothelial cell area (cells comprising the walls of blood vessels) occupied by NP. Values are means with standard deviations. *indicates significantly different (*P* < 0.05) than interstitial area of all other groups. ^+^indicates significantly different (*P* < 0.05) than endothelial cell area of all other groups. (b) The delivery of FGF-2 bearing nanoparticles by ultrasonic microbubble destruction elicits arteriogenic remodeling in gracilis adductor muscle. (A)–(D) Representative whole-mount images of fluorescently labeled SM *α*-actin+ vessels in gracilis adductor muscles 7 and 14 days after FGF-2 (A) and (B) and BSA (C) and (D) treatment. Note the significant increase in arteriolar caliber and density in FGF-2-treated muscles. (E) Bar graph of arteriole line intersections at both time points for FGF-2, BSA, and sham surgery treatment. Values are means with standard errors. *indicates significantly different (*P *< 0.05) than BSA and sham surgery at day 14. Reprinted from [57] with permission from Wiley.

Another example of combining NP with MB to enhance gene delivery is shown in Figure 6. This study showed that gene delivery of recombinant growth factors to stimulate arteriogenesis is possible through a combination of NP, an albumin-based MB contrast-agent, and US *in vivo* (Figure 6(a)) [57]. After verifying that ultrasonic MB destruction effectively deposited intravascular polystyrene nanoparticles into mouse adductor skeletal muscle, FGF-2-bearing biodegradable PLGA NPs (FGF-2-NP) were generated and coadministered intraarterially with MB, and delivery was spatially targeted to ischemic mouse hind limbs using 1 MHz US. The delivery of FGF2-NP stimulated appreciable arteriogenic remodeling in ischemic mouse hind-limb adductor muscles. This response included an increase in the total number of large and moderate diameter arterioles (i.e., >15 *μ*m in diameter), as well as a marked luminal expansion of both collateral and transverse arterioles (Figure 6(b)) two weeks after treatment. This system efficiently delivered PLGA FGF2-NP to mouse muscle in a model of hind-limb arterial insufficiency. This method has several features that may enhance its potential for successful clinical translation, including minimally invasive targeting, sustained growth-factor delivery, and retention of growth factor bioactivity. Ultimately, these results indicate that ultrasonic MB destruction has potential as a platform for therapeutic delivery of NP *in vivo* for vascular remodeling, and depending on antitumor therapeutics chosen, this may have important implications also for tumor therapy using cytokine gene delivery, for example.

#### 3.1.5. Future Formulations: Promise for Echogenic PEGylated or Dendrimer PLGA Formulations

**Figure 7:**
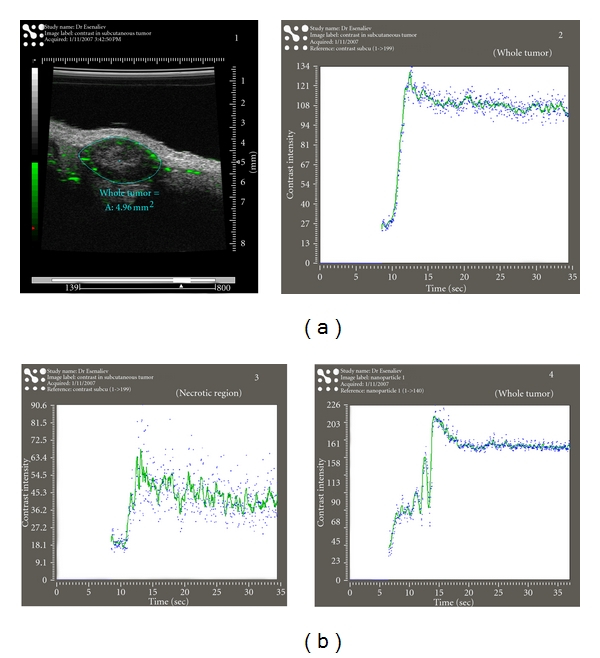
. Echogenic PLGA nanoparticles can be utilized also as ultrasound contrast agents *in vivo. *(a) (1) A tumor image obtained with the high-resolution ultrasound system VEVO770 (VisualSonics). (2) Kinetics of the contrast agent in the whole tumor shown in (1). (b) (3) Kinetics of the contrast agent in the central area of the tumor shown in (1). (4) Kinetics of the PLGA nanoparticles in the whole tumor shown in (1).

As we have shown, PLGA NP can be echogenic and serve as a contrast agent in addition to as a gene delivery vehicle. For example, *in vivo* ultrasound imaging can be accomplished with a high-resolution small imaging system apparatus and is illustrated in Figure 7. We show an example of US imaging for examining the kinetics of PLGA NP *in vivo* (prostate tumors) by using novel, high-resolution ultrasound imaging system Vevo 770 developed by VisualSonics (Toronto, Canada). The system has the ability to visualize and quantify tumors, hemodynamics, and therapeutic interventions with resolution down to 30 microns noninvasively and in real time.  Figure 7(a) shows an image of a DU145 prostate tumor in a nude mouse obtained with the system following intravenous administration of PLGA NP (same NP as described in Figure  4(b)). The system was capable of detecting the distribution of an unlabeled ultrasound contrast agent (UCA, VisualSonics) and allowed its visualization in the tumor (the areas with high concentration are represented in green). A specially developed computer code allowed to quantify kinetics of this UCA in the tumor (Figure 7(a), right panel). There was a sharp increase of the concentration in the whole tumor within first 2 to 3 seconds after the injection that was followed by a wash-out process (decrease of the contrast intensity). The necrotic areas at the center of the tumor had similar kinetics but less concentration of the UCA due to lower vascularization (Figure 7(b), left panel). In contrast, injection of the PLGA nanoparticles into the same mouse (after clearance from the UCA) demonstrated almost constant concentration of the PLGA nanoparticles 15 seconds after the injection (Figure 7(b) -(2)). This effect resulted from competition of two processes: (1) the decrease of nanoparticles concentration in blood and (2) the increase of their concentration in the tumor blood vessels due to the EPR effect. Moreover, the contrast intensity produced by the PLGA nanoparticles (~175) was much higher compared to that of the UCA (~100). These data indicate that high-resolution ultrasound small animals imaging systems are able to detect the PLGA nanoparticles in tumors *in vivo* and that these nanoparticles are highly echogenic.

**Figure 8:**
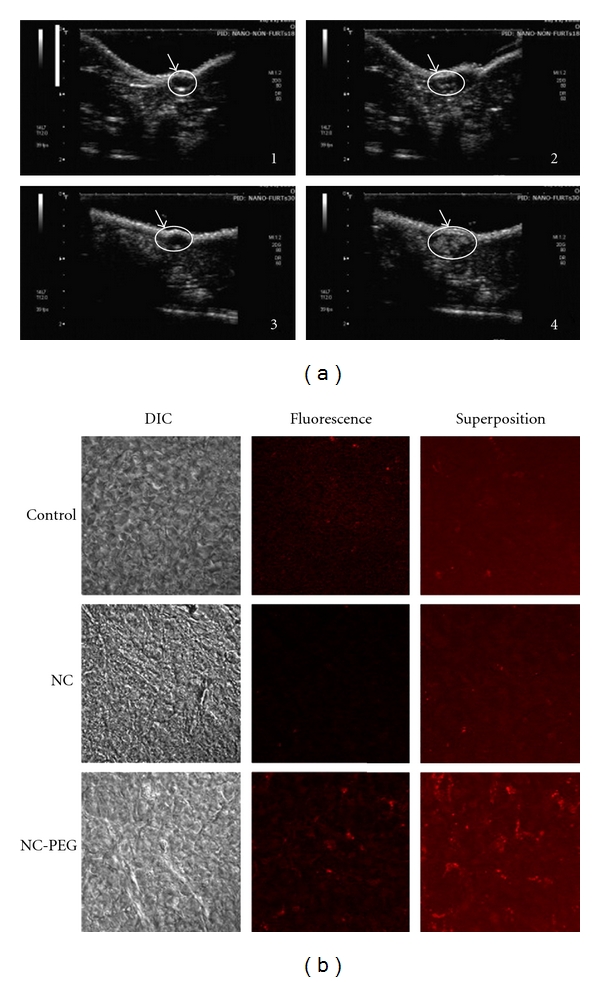
PEG-PLGA particles as ultrasound contrast agents *in vivo.* (a) Ultrasound images of mouse pancreatic tumors obtained in a nonlinear imaging mode before injection (1)–(3) and after intratumoral injection of plain nanocapsules (2) or PEGylated nanocapsules (4). The tumor is indicated as the region of interest (ROI) represented by a circle. (b) Confocal microscopy images of tumor slices from a control mouse (control) and mice after 24 h of an intravenous injection of non-PEGylated nanocapsules (NC) and PEGylated nanocapsules (NCL-PEG). DIC corresponds to differential interference Nomarski contrast. Red fluorescence corresponds to PLGA dyed with Nil Red. Reprinted from [58] with permission from Elsevier.

Further modifications can be made to echogenic PLGA NP to enhance their potential for longer circulation half-life and for enabling tumor-specific targeting. For example, surface modifications can be made to polymeric nanoparticles to add PEGylated phospholipids in order to escape recognition and clearance by the mononuclear phagocyte system and achieve passive tumor targeting. Nanoparticles consisting of a shell of PLGA encapsulating a liquid core of perfluorooctyl bromide (PFOB) can be decorated with poly(ethylene glycol-2000)-grafted distearoylphosphatidylethanolamine (DSPE-PEG) and resulting particles still are echogenic and can allow visualization of MIA-PaCa-2 pancreatic tumors *in vivo,* following intratumoral or intravenous injection (Figure 8(a)). In this example, the tumor was visualized only following intratumoral UCA injection. Despite the absence of echogenic signal in the tumor after intravenous injection of NP, histological analysis revealed their accumulation within the tumor [58], and this accumulation can be explained by their increased circulation time due to their PEGylated surface (Figure 8(b)). PEG coating protects NC-PEG against plasma protein adsorption and therefore against recognition by phagocytic cells. The increased circulation time favors their passive targeting in tumor tissue by the enhanced permeation and retention effect [59]. A quantitative biodistribution of NC-PEG likely would have been helpful to assessing their actual concentration in tumors and determining the concentration threshold necessary for ultrasonography with these new UCAs.

## 4. Novel Directions

### 4.1. PLGA as an Ultrasound Contrast Agent

 Other UCAs recently developed by Nestor et al. include air-filled nanocapsules made of PLGA. These have a critical advantage over current commercial UCAs, which are not capable of penetrating the irregular tumor vasculature due to their larger dimensions. These new nanoscale UCAs based on PLGA can therefore be used to enhance tumor detection since they display enhanced stability compared to commercially available UCAs when in the presence of US. Air-filled nanocapsules with a mean diameter of ~370 nm have been shown to maintain a spherical shape and thickness <50 nm and remain echogenic [60], providing an enhancement of up to 15 dB at a concentration of 0.045 mg/mL at a frequency of 10 MHz. Loss of signal for air-filled nanocapsules was 2 dB after 30 min, suggesting high stability. This UCA therefore has the potential to be applied to ultrasound imaging. Other NPs that are in development as UCAs include polymer-based multifunctional nanoparticles that exhibit a near-infrared absorption and can be used as a novel photoacoustic contrast system [61, 62]. Photoacoustics is a new imaging modality in which laser light is shined into tissue and adsorbed by inherent or synthetic molecules or particles and generates ultrasound. Submicron-sized NPs with a high encapsulation efficiency have been created by the incorporation of near-infrared (NIR) dyes in PLGA via a spray-drying process. Subsequent centrifugation yielded two size fractions ranging from ~445–550 nm to ~253–305 nm in diameter [61, 62]. These NIR PLGA NP exhibited photoacoustic properties using an Nd:YAG laser-based system but did not show any detrimental effects on cell viability or mitochondrial activity. Photoacoustics properties persisted in cell culture for up to 2 days, suggesting the excellent photoacoustic properties plus the low cytotoxicity profile renders these dye-loaded PLGA particles promising candidates for a resorbable photoacoustic contrast system *in vivo*.

### 4.2. The Future for Biodegradable PLGA for Gene Delivery

#### 4.2.1. Developing Better PLGA Nanoparticles

One improvement that might impact PLGA NP effectiveness as a gene delivery agent *in vivo* is to improve the acidic microclimate developed during polymer degradation which can potentially damage the nucleic acid that may be encapsulated or complexed to the NP. Buffering agents have been used that incorporate antacid (0, 3% MgOH_2_) [63], whereby PLGA microspheres maintained a more homogeneous surface, resulting in a significant reduction of the commonly seen “burst effect.” For example, PLGA microspheres of ~47 micron have been shown to completely release pDNA over the course of two months, addressing some of the major problems associated with DNA encapsulation and release. We envision that these same buffering principles might be applicable to smaller PLGA particles to help reduce any pDNA degradation that might occur secondary to polymer degradation prior to or following US-mediated gene delivery *in vivo*.

#### 4.2.2. Current New Technology: Nonechogenic PLGA NP Have Been Used with Success for Targeted Drug Delivery

**Figure 9:**
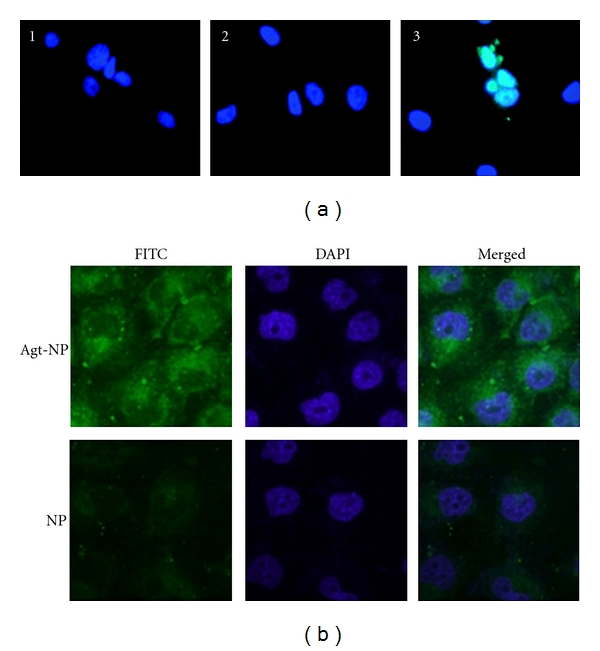
Targeted nanoparticles are promising for future *in vivo* gene delivery approaches. (a) PSMA-targeted PLGA-based microparticles enter LNCaP (PSMA+) PCa cells. Untreated control (1), after 30 min of exposure to nontargeted FITC-loaded (2), and targeted FITC-loaded (3) MBs. Cell nuclei were stained with Hoechst (blue). The number of green-positive cells per field was significantly different from that of nontargeted MBs. Reprinted from [64] with permission from American Chemical Society. (b) Confocal fluorescent scanning microscopy images detecting cellular uptake of MUC-1 targeted Aptamer conjugated NPs (top row) or NPs (bottom row) in MCF-7 cells. Green fluorescent FITC was encapsulated in Apt-NPs and NPs. The nuclei were stained blue with DAPI. The right column showed the merged images of the FITC and the DAPI channels. MCF-7 cells were exposed to FITC-encapsulated Apt-NPs or NPs at 100 *μ*g/mL for 2 hours. Reprinted from [66] under the terms of the Creative Commons Attribution License.

Several studies have reported the use of PLGA NP or MP for targeting drug delivery to tumor cells. These PLGA NPs are still under development and are not echogenic. Thus, these new approaches will be useful when adapted for the field of ultrasound-mediated gene delivery. We envision that the same targeting moieties can be conjugated or complexed onto PLGA particles with acoustic activity for future applications to gene delivery by sonoporation. We describe here examples of targeting using PLGA NP, including the studies described in Figure 8. In Figure 9(a), PLGA-based MPs were produced that were able to target prostate tumor cells expressing the prostate-specific membrane antigen or PSMA [64]. A set of air-filled MBs of various biocompatible polymer compositions were prepared and characterized in terms of morphology and echogenic properties after exposure to US. MBs derived from PLG-poly(ethylene glycol) (PEG) copolymer resulted in being the most effective in terms of reflectivity. PLGA-PEG was therefore preconjugated before MB preparation with an urea-based PSMA inhibitor [65]. Using this copolymer as a starting material, the MBs were examined *in vitro* for their targeting efficacy toward PSMA-positive cells. Fluorescence microscopy showed a specific and efficient adhesion of targeted MBs to LNCaP cells. This model for targeting PSMA might be further optimized for smaller particle use (echogenic nanoparticles) and used for prostate cancer diagnosis and drug or gene delivery.

**Figure 10:**
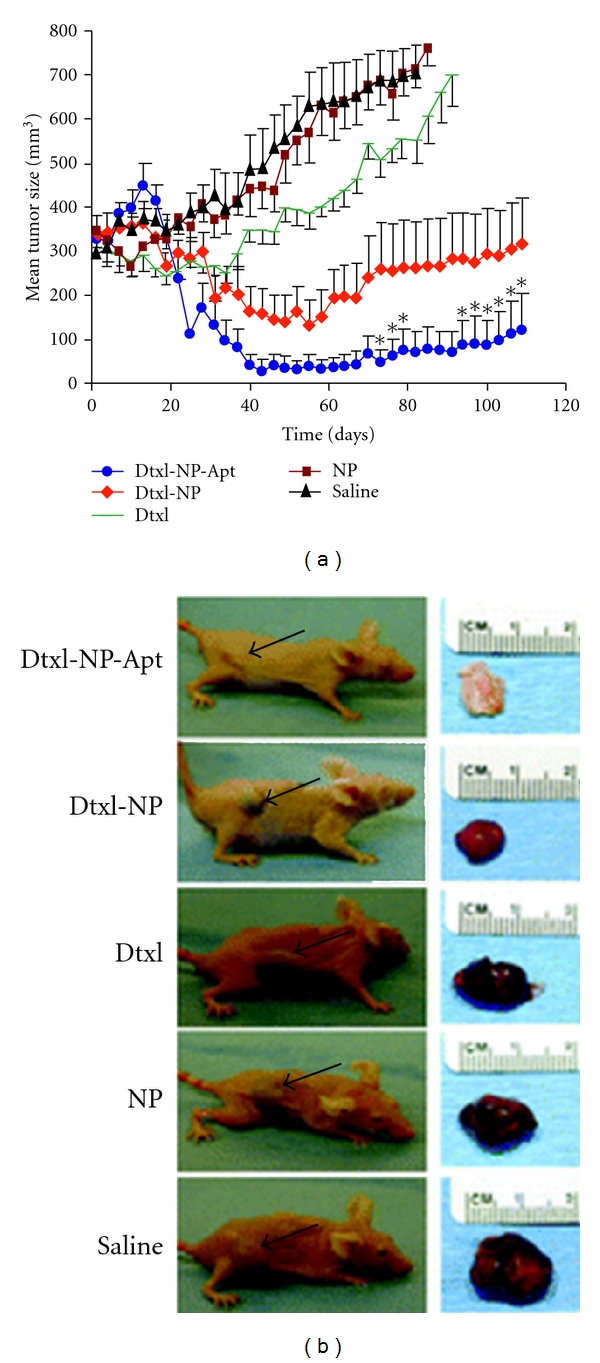
Future Potential of PLGA-based nanoparticles for realizing efficient *in vivo* drug delivery. (a) PLGA formulations for drug delivery. The antitumor efficacy of single intratumoral injections of drugs or controls was compared for several NP groups. Groups examined included saline, pegylated PLGA NP (NP), Docatexel- (Dtxl-) encapsulated NP (Dtxl-NP) at 40 mg/kg, or Dtxl-NP-PSMA targeted Aptamer conjugates at 40 mg/kg (Dtxl-NP-Apt). Aptamer-targeted NPs were more efficacious in tumor reduction compared to control groups. Data points labeled with “*” were statistically significant compared with all other groups by analysis of variance (ANOVA) at a 95% confidence interval. (b) Representative mice at the end point for each group are shown (left) alongside images of excised tumors (right). For the Dtxl-NP-Apt group, which achieved complete tumor regression, the scar tissue and underlying skin at the site of injection are shown. Black arrows point to the position of the implanted tumor on each mouse. Reprinted from [68] with permission from PNAS.

Additional targeting moieties for PLGA NP have utilized aptamers, which are single-stranded RNA or DNA oligonucleotides ~15–60 bp in length that can bind with high affinity to specific molecular targets. Most aptamers to proteins bind with a Kd of ~1 pM to 1 nM, which is an affinity level similar to that of monoclonal antibodies. Moreover, aptamers are able to bind to nucleic acid, proteins, and small organic compounds and enable targeting to specific cells, in a manner similar to the concept of high-affinity antibodies. For example, a targeting nanoparticle was developed that had a mucin-1- (MUC-1-) specific Aptamer (Apt-NP) conjugated to the surface (Figure 9(b)). MUC1 protein is an attractive target for anticancer drug delivery owing to its overexpression in most adenocarcinomas. In this study, a reported MUC1 protein aptamer was exploited to target Paclitaxel- (PTX-) loaded PLGA NPs of ~225.3 nm in size. Using MCF-7 breast cancer cells as a MUC1-overexpressing model, the aptamer increased the uptake of nanoparticles into the target cells as measured by flow cytometry. Moreover, the PTX-loaded Apt-NPs enhanced *in vitro* drug delivery and cytotoxicity to MUC1+ cancer cells, as compared with nontargeted NP lacking the MUC1 aptamer. The behavior of this novel aptamer-NP bioconjugate suggests that MUC1 aptamers may have a wider application potential in targeted gene delivery towards MUC1-overexpressing tumors [66]. Other aptamers used for targeted delivery of NP have included PLGA conjugated to polyethylene glycol (PEG), which have been used to deliver encapsulated prodrugs. PLGA NP are targeted using aptamers with affinity for the extracellular domain of PSMA [67, 68]. Such NP are highly efficacious compared to prodrugs *in vivo,* and pharmacokinetic studies showed improvements in tolerability and efficacy compared to standard chemotherapy (Figure 10). We envision that such a NP design might greatly enhance gene delivery targeted specifically to prostate cancer cells expressing PSMA.

Other uses of aptamers have included a PLGA NP of ~156 nm decorated with aptamer AS1411 (Apt-NP) [69]. AS1411 is a DNA aptamer that specifically binds to nucleolin, a protein upregulated in the plasmsa membrane of both cancer cells and angiogenic blood vessels. Apt-NP was used to facilitate antiglioma delivery of paclitaxel (PTX). The Ap-nucleolin interaction significantly enhanced cellular association of nanoparticles in C6 glioma cells and increased the cytotoxicity of its payload. Prolonged circulation and enhanced PTX accumulation at the tumor site were achieved by Ap-PTX-NP, which also yielded higher tumor inhibition on C6 glioma xenografts and prolonged survival when comapred to PTX-NP (untargeted) and Taxol. Therefore, aptamer-functionalized PLGA NP can be an efficient therapeutic and this design might be adapted as well for successful potential gene delivery to glioma.

*Antibodies*. Other PLGA NP that have been used for effective cellular targeting have included PLGA nanobubbles (NB) for cancer targeting and imaging using optical and US modalities. For example, PLGA NBs have been conjugated with cancer-targeting ligands such as a humanized antibody to target the overexpressed TAG-72 antigen [70]. NB-assisted dual-mode imaging was demonstrated on a gelatin phantom with multiple embedded tumor simulators at different NB concentrations, demonstrating the feasibility of using dual-mode contrast agents for cancer targeting and simultaneous fluorescence/US imaging. Another PLGA-PEG NP recently described coupled the J591 monoclonal antibody to its surface in order to direct targeting towards PSMA-expressing prostate cancer cells. A pDNA encoding *β*-gal was complexed to this NP via a salicyl-hydroxamic-acid- (SHA-) derivatized PEI. After encapsulation, an 8- to 10-fold enhancement in gene expression was attained due to enhanced specific internalization and uptake of the complex in PSMA-expressing cells. The release of pDNA from NP showed a small initial burst release followed by a 5% release over 48 h. The release accelerated thereafter and ~60% was released within a month. Also, the PEG-PLGA composition (triblock polymer) was found to enhance the polyplex/microparticle localization to the cell nucleus and this enhanced the endocytic process of J591-mediated targeting in prostate cancer cells.

*RGD*. Another class of polymeric contrast agents with targeting potential was described in which the Arg-Gly-Asp (RGD) peptide sequence was conjugated to either PLA or PLGA microcapsules [72, 73]. These hollow, biodegradable microcapsules targeted *α*v*β*3 and *α*v*β*5 integrins, typically expressed during angiogenesis. *In vitro* results indicated that the modified capsules remained echogenic and adhered specifically to the breast cancer cell line MDA-MB-231. An interesting modification of this approach has been utilization of a cyclic RGD targeting moiety conjugated via a micelle-type PLGA-4 arm-PEG branched polymer for detecting and treating pancreatic cancer [74]. These NPs contained the 4-arm PEG as a corona and PLGA as a core, while the particle surface was conjugated with cRGD for *in vivo* tumor targeting. The hydrodynamic size of NP was ~150–180 nm and NIR microscopy and flow cytometry studies showed that the cRGD-conjugated NPs were taken up more efficiently by U87MG glioma cells overexpressing integrins. Whole-body imaging showed that the cRGD NP had the highest accumulation in pancreatic tumors at 48 h after-injection with low *in vivo* toxicity. We would predict additional receptor targeting will be attempted in the near future and this will likely extend targeting of PLGA nanoparticles to the VEGFR and EGFR family of receptors to achieve enhanced drug and gene delivery, as already has been shown to work for microbubbles targeting the VEGFR2 receptor in tumor-associated endothelial cells [75, 76].

*Proapoptotic.* PLGA NPs coated with a proapoptotic monoclonal antibody have been efficient in delivering drugs in a targeted manner. For example, use of NP coated with Conatumumab or AMG 655 death-receptor 5 antibodies (DR5-NP) has preferentially targeted DR5-expressing cells and has induced apoptosis in a specific manner while also delivering encapsulated drugs such as camptothecin [77]. This is an interesting example of antibody conjugation to NP surface that can be exploited for the dual functions of targeted drug delivery and cell killing. Another example used gene delivery to achieve apoptosis in prostate tumors by delivering pDNA expressing an shRNA against annexin A2 [78]. In prostate cancer progression, annexin A2 is upregulated cancer. These PLGA NP sustained intracellular delivery of shRNA and achieved long-term downregulation of annexin A2. Intratumoral administration of pDNA-shAnxA2-loaded NP to xenograft prostate tumors in nude mice inhibited tumor growth through reductions in annexin A2 and VEGF levels. This interesting study suggests that the use of sustained-release polymeric NP for delivering shRNA constructs might serve as an effective adjuvant treatment option for cancer.

 One important final consideration for practical use of PLGA or any NP for receptor and other tumor-targeted genes delivery is the size range required for therapeutically effective drug concentrations at tumor sites while reducing undesirable side effects. For example, targeted drug delivery using long-circulating particulate drug carriers of controlled size (<100 nm diameter) (reviewed in [79]) holds great potential to improve the treatment of cancer by selectively providing enhanced permeability and retention (EPR) and optimal tumor distribution of NP.

### 4.3. Future Uses: Targeted Echogenic PLGA Nanoparticles for Theranostic Applications

For future applications, echogenic PLGA NP will be important to achieving theranostic applications (diagnostic and therapeutic) for cancer. For example, for early cancer diagnosis and therapy, new systems will be continually designed and developed with key components uniquely structured at nanoscale according to medical requirements. For imaging, it is envisioned that quantum dots with emissions in the near-infrared (NIR) range will continue to be utilized for delivering drugs and/or nucleic acids. For example, quantum dots have been successfully conjugated onto a surface of a nanocomposite material consisting of a spherical polystyrene matrix (<150 nm). Internally embedded supraparamagnetic Fe_3_O_4_ nanoparticles (<10 nm) could be successfully loaded with PTX onto this nanocomposite material by using a layer of PLGA [80]. Variations of such a nanocarrier were then successfully conjugated to antibodies or aptamers to achieve cell-specific targeting. For example, these PTX-loaded PLGA nanocarriers were conjugated to an anti-PSMA antibody for targeting of LNCaP prostate tumors with high specificity* in vivo*. For diagnostic applications, we envision that nanoparticle contrast agents will become of increasing interest for high-resolution imaging in medicine. For example, a novel dual-modal contrast agent has been developed for structural and functional imaging of cancer [81]. This contrast agent was fabricated by encapsulating indocyanine green (ICG) in PLGA MB. The technical feasibility of concurrent structural and functional imaging was demonstrated through a series of tests in which an aqueous suspension of ICG-PLGA MB was injected into a transparent tube embedded in an Intralipid phantom at different flow rates and concentrations. Concurrent fluorescence imaging and B-mode ultrasound imaging successfully captured the changes of microbubble flow rate and concentration with high linearity and accuracy. One potential application of the proposed ICG-PLGA MB would be for the identification and characterization of peritumoral neovasculature. Enhanced coregistration between tumor structural and functional boundaries could be achieved using US-guided near-infrared diffuse optical tomography. In a similar manner, photoacoustic imaging applications also will be implemented, for example, NP exhibiting a near-infrared (NIR) absorption can be prepared by incorporation of ICG into PLGA [61, 62]. These NPs were biocompatible *in vitro* and had a high NIR dye encapsulation efficiency (>98%) and two different size fractions were obtained of ~640 nm and ~390 nm. Cytotoxicity studies indicated no changes in metabolic activity, proliferation, or membrane integrity. Their high optical absorption at ~800 nm in combination with absence of cytotoxicity qualifies the ICG-PLGA particles as promising candidates for degradable photoacoustic contrast agents in future studies. 

Other nanoparticles in development include composite PLGA-magnetic particles for simultaneous drug delivery and imaging [82], and these might also be applied to gene delivery in future applications. These magnetic nanoparticles were embedded in PLGA matrices (PLGA-MNP) to achieve a dual-drug delivery and imaging system and were capable of encapsulating both hydrophobic and hydrophilic drugs in a 2 : 1 ratio while retaining favorable biocompatibility and cellular uptake properties. For targeted delivery of drugs, targeting ligands such as Herceptin were tested, demonstrating enhanced cellular uptake. Also, magnetic resonance imaging was used to show improved contrast by PLGA-MNP compared to commercial contrast agents due to higher T2 relaxivity with a blood circulation half-life of ~47 min in a rat model. These PLGA-based matrices may be applied to both imaging and adapted to achieve successful gene delivery.

## 5. Conclusions

PLGA and other nanoparticle delivery systems in general have distinct advantages for gene delivery, such as protecting DNA from degradation and enhancing complex stability. PLGA-based NPs can penetrate deeply into tissues through fine capillaries and are generally taken up efficiently by cells. This allows efficient delivery and accumulation of therapeutic agents, such as conventional medicines, vaccine antigens, proteins, and genes, to target sites (tissues or organs) in the body. PLGA NPs also have the advantage of sustained and controlled release of the encapsulated therapeutic agent over a period of days to several weeks compared with natural polymers, which have a relatively short duration of drug release. PLGA and other NP, if synthesized in a manner to render acoustic activity, can strongly promote not only therapy delivery but also serve as contrast agents for standard US-mediated imaging or photoacoustic imaging. PLGA NP will continue to be refined and improved also to target gene and drug delivery to certain cells and tissues via conjugation of highly specific antibodies, aptamers, or other molecules to their surface. For gene delivery, other nucleic acid types will be expanded either loaded onto or into PLGA NP, including promising directions using siRNA/miRNA technology to silence multiple tumor-promoting genes, for example.

Overall, the promise of these technologies and approaches using PLGA NPs represents a novel and potentially more effective means to manage cancer and other diseases. However, thorough evaluation for pharmacokinetics, biodistribution, toxicity, and efficacy of particular therapeutic agents (gene or drugs) is still required before widespread use will be achieved for PLGA NP in clinical trials. Nevertheless, gene delivery using PLGA- or PLGA-based polymers is an attractive area with vast opportunities for biomedical research. During the past few years, research on PLGA NP has increased in the field of drug delivery and targeting of NP to cancer cells or blood vessels within tumors. We predict these improvements also may promote advances in the gene delivery applications of PLGA NP. These polymers are increasingly becoming feasible candidates for delivering nucleic acids as anticancer agents and for vaccine immunotherapy. We also believe that PLGA-based NP will be developed further to enable treatment and diagnosis of a variety of other diseases besides cancer. Therefore, our predictions are that PLGA-related NP technology should play increasingly more important and mainstream roles in tissue engineering and in other emerging areas such as stem cell research.
